# Assessing anger regulation in middle childhood: development and validation of a behavioral observation measure

**DOI:** 10.3389/fpsyg.2015.00453

**Published:** 2015-04-24

**Authors:** Helena L. Rohlf, Barbara Krahé

**Affiliations:** Department of Psychology, University of PotsdamPotsdam, Germany

**Keywords:** anger regulation, middle childhood, behavioral observation, aggression, social rejection

## Abstract

An observational measure of anger regulation in middle childhood was developed that facilitated the *in situ* assessment of five maladaptive regulation strategies in response to an anger-eliciting task. 599 children aged 6–10 years (*M* = 8.12, *SD* = 0.92) participated in the study. Construct validity of the measure was examined through correlations with parent- and self-reports of anger regulation and anger reactivity. Criterion validity was established through links with teacher-rated aggression and social rejection measured by parent-, teacher-, and self-reports. The observational measure correlated significantly with parent- and self-reports of anger reactivity, whereas it was unrelated to parent- and self-reports of anger regulation. It also made a unique contribution to predicting aggression and social rejection.

## Introduction

Anger is a common emotion in childhood. School-aged children have reported feeling angry once a day on average and more often described their anger intensity as strong than as moderate or low (von Salisch, 2000). Anger may be defined as “the appraisal that a goal of personal significance has been blocked and readiness to act with increased effort to overcome obstacles and achieve the goal” (Cole, 2014, p. 204). A large body of research has shown that deficits in anger regulation are related to various problematic outcomes in childhood, including aggression and peer rejection (see Lemerise and Harper, 2010; Röll et al., 2012, for reviews). Given this great importance of anger regulation skills for children's social functioning (Fabes and Eisenberg, 1992), it is essential to have valid methods for the assessment of anger regulation strategies in childhood. The present study was conducted to develop and validate an observational method for assessing anger regulation in middle childhood in response to an anger-eliciting task.

According to Gross (1998), emotion regulation is defined as “the processes by which individuals influence which emotions they have, when they have them, and how they experience and express these emotions” (p. 275). Emotion regulation includes attentional, cognitive, and behavioral attempts to manage the internal experience or the external expression of emotion (Eisenberg and Spinrad, 2004). The development of emotion regulation skills makes major progress throughout childhood (Lemerise and Harper, 2010). By the time they start school, most children have developed a set of strategies that enable them to regulate their emotions, and they have also understood that the external expression of emotions does not have to match the internal emotional experience (Saarni and von Salisch, 1993). They show an increasing use of strategies for regulating the anger expression (e.g., by substituting or neutralizing the anger expression) in order to comply with cultural display rules for the expression of emotions (Zeman and Garber, 1996). However, there is evidence that children find the regulation of anger more difficult than the regulation of other negative emotions. In a study by Zeman and Shipman (1997) children reported a lower self-efficacy regarding the regulation of the expression of anger compared to the regulation of the expression of sadness. Similarly, Waters and Thompson (2014) found that children perceived the regulation of anger as more difficult than the regulation of sadness. In addition, their study revealed that children perceive different strategies to be more effective in regulating anger compared to sadness. Notably, children rated *problem-solving behavior* to be more effective in managing the experience of anger, whereas the strategies *seeking social support* and *venting the emotion* were seen as more effective in regulating sadness. These results are in line with the theoretical conceptualization of anger as a response to the blockage of a goal: As a strategy that is directed at removing the obstacle to goal attainment, problem-solving is more likely to effectively reduce anger than strategies that focus on the emotion experience.

Although the majority of the studies on anger regulation in middle childhood have relied on parent- and self-reports of anger regulation, there are several concerns about the use of such measures. With regard to self-reports, thinking and talking about complex processes such as emotion regulation requires an appropriate level of cognitive and linguistic skills that might not have developed sufficiently at this age. Furthermore, even if a child is able to generate strategies for regulating emotional states, it remains questionable whether children's self-reports on how they might behave correspond to their behavior in a real emotion-evoking situation (Underwood, 1997b). Regarding anger in particular, children's reports may be distorted as anger is related to an impulse to act and has been shown to narrow attention, bias judgments, and influence information processing (Litvak et al., 2010). These characteristics make it difficult to behave in a reflected way in the state of anger. Thus, children who theoretically know about adaptive regulation strategies may have difficulties acting according to this knowledge when they are angry. A study by Parker et al. (2001) showed that 2nd grade children's reports about how they would express their anger in a hypothetical scenario differed substantially from their behavior in a live situation. In the live context, children reported feeling less anger, expressed less anger, and dissembled their anger more. Furthermore, the children generated fewer strategies for hiding their anger in the live context in comparison to the hypothetical context. Based on these results, the authors warned that children's self-reports in response to hypothetical vignettes should not be considered representative of their actual behavior in live situations.

Parents' ratings may provide more valid information about their children's anger regulation skills, as they have the opportunity to observe their children in anger-arousing situations. Parents, however, can only give information about their children's behavior in the family context. The emotion-related behavior children show in their family cannot easily be generalized to behavior in other contexts, such as the school. Children have reported controlling their expression of emotion significantly more in the presence of peers compared to parents (Zeman and Garber, 1996). This discrepancy might be particularly large with respect to anger as children anticipate greater negative social consequences from peers in response to displaying anger compared to other emotions (Underwood, 1997a).

These findings suggest that an observation of the children's behavior in an anger-eliciting situation might provide a better assessment of anger regulation strategies than parent- or self-reports. By recording anger regulation skills *in situ*, behavioral observations may yield more ecologically valid conclusions about anger regulation skills than self- and parent-reports. To date, observational measures of anger regulation have been primarily used in studies with children of pre-school age. For example, Tan et al. (2013) developed a paradigm in which children aged between 24 and 48 months were made to wait for a desired gift while playing with a boring toy. Two adaptive anger regulation strategies, distraction and calm bids, were identified and were found to be negatively linked to difficulties in child temperament (negative affectivity and low effortful control). The use of behavioral observation measures in studies with preschoolers is often based on the argument that the use of self-reports is not possible due to the limited cognitive abilities of children at this age (e.g., Helmsen and Petermann, 2010). The results of the study of Parker et al. (2001) described above indicate that the same reasoning can be applied to school age children. However, when conducting behavioral observations in middle childhood, it is crucial to know how valid the obtained data of anger regulation actually is and whether observational measures can add additional information beyond parent- or self-reports. In our study we addressed this question by assessing anger regulation through behavioral observation as well as parent- and self-reports and by examining the associations of these different methods with aggression and social rejection. This enabled us to examine if the observational measure can explain unique variance of these two outcomes. To our knowledge, there are no studies to date that have directly addressed this issue.

Emotion regulation is not limited to successful, adaptive regulation strategies but also includes maladaptive strategies (Eisenberg and Spinrad, 2004). However, regulation strategies are not generally good or bad, as their adaptivity can vary across different contexts (Gross, 1998). Thus, strategies can have different consequences depending on the situation in which they are used and depending on characteristics of the person who uses them, such as age and gender. Therefore, in the present study we defined the adaptivity of the anger regulation strategies specifically in terms of their consequences on aggression and social rejection. Accordingly, our classification into adaptive and maladaptive strategies was based on studies that investigated the associations of anger regulation strategies with aggression and social rejection. With regard to aggression, it has been found that in frustrating situations aggressive children more often focus on the frustrating stimuli, show more external regulation (e.g., swearing or handling the task material roughly), and show a higher tendency to resign from the situational demands than do non-aggressive children (Melnick and Hinshaw, 2000; Gilliom et al., 2002; Crockenberg et al., 2008; Helmsen and Petermann, 2010). In contrast to these maladaptive forms of anger regulation, the ability to distract oneself from the source of frustration and the use of problem-oriented behavior has been found to be used more often by non-aggressive children (Orobio de Castro et al., 2005). With regard to the application of display rules about the socially acceptable expression of anger, there is evidence that non-aggressive children use display rule strategies for regulating the expression of anger more often compared to aggressive children (Underwood et al., 1992; Cole et al., 1994).

Similar findings have been obtained with regard to the link between anger regulation strategies and social rejection. Focusing on negative aspects of a frustrating task, showing less use of active distraction from a frustrating stimulus, and showing less use of display rule strategies could be identified as predictors of low social preference and social rejection (McDowell et al., 2000; Melnick and Hinshaw, 2000; Trentacosta and Shaw, 2009), respectively. Furthermore, socially rejected children have been found to express their anger more compared to their socially accepted peers (Dearing et al., 2002). Based on these results, we distinguished seven observable strategies of anger regulation: The strategies *visual focus, verbal focus, venting the anger*, and *resignation* were conceptualized as maladaptive, whereas *distraction, solution-orientation*, and *the use of display rule strategies* were defined as adaptive in terms of aggression and social rejection. With regard to the strategy *venting the anger*, it is important to note that this behavior is not consistently conceptualized as a regulation strategy but sometimes seen as the simple expression of the anger experience that has no regulatory function. Different authors have conceptualized anger expression and anger regulation as distinct constructs and have considered anger expression as the outcome of the regulation process or as an indicator of anger reactivity (Melnick and Hinshaw, 2000; Dearing et al., 2002). However, as we assume that such behavior includes the attempt to reduce the anger intensity, in line with other authors (Grob and Smolenski, 2005; Helmsen and Petermann, 2010), we consider external anger-related behavior, such as venting the anger, as part of anger regulation.

A further important emotion regulation strategy in childhood is *seeking social support*. Whether this strategy is adaptive or maladaptive depends on the likelihood that social support may be obtained. Research has shown that help-seeking behavior is a mediator between insecure attachment style and maladjustment (Larose and Bernier, 2001) and that seeking social support during frustrating situations effectively reduces anger in children and adolescents (Spangler and Zimmermann, 2014). However, these links have been studied in situations where supportive others were available, for example in the form of emotional support provided by mothers. In our paradigm, children encountered the anger-eliciting task in the presence of a stranger who was instructed not to respond to requests for help. If children looked at the experimenters, they did not respond, if they directly asked for help, they were told they had to manage the task on their own. In this context, repeated attempts at securing social support, despite having noticed that no help can be expected, is not considered an adaptive strategy. Consistent with this reasoning, studies that observed children in a frustrating situation in which social support was not available or only to a limited degree, did not find associations between the strategy *seeking support* and aggression (Gilliom et al., 2002; Helmsen and Petermann, 2010). Thus, in line with the classification of regulation strategies by other authors (Grob and Smolenski, 2005), we considered this strategy to be neither adaptive nor maladaptive in our behavioral observation measure, although it may well be adaptive in other contexts in which support is actually available. To highlight this point, we refer to this category as *ineffective help-seeking* in the context of our methodological approach.

The aim of the present study was to develop and validate a method for assessing anger regulation in children through behavioral observation in an anger-eliciting situation. The measure was designed to meet two objectives: (a) to identify anger regulation strategies defined as maladaptive with regard to social rejection and aggression that are open to observation, and (b) to categorize any additional strategies in response to the anger-eliciting task to provide a comprehensive description of the children's behavioral strategies of dealing with their anger. Anger was induced through a frustration, defined as the blocking of a goal-directed activity, by presenting the children with an unsolvable task, as described in the Methods section below. A coding system of children's behavior during completion of the task facilitated the identification of the adaptive and maladaptive regulation strategies as well as additional strategies that were part of the children's behavioral repertoire in dealing with their anger during the task. The coding system was based on several studies which have used a similar approach for categorizing emotion regulation strategies (Fabes and Eisenberg, 1992; Melnick and Hinshaw, 2000; Gilliom et al., 2002; Helmsen and Petermann, 2010), and on other work addressing emotion regulation in children (Grob and Smolenski, 2005; Petermann and Wiedebusch, 2008).

Construct validity was assessed by correlating the behavioral measure with parent- and self-reports of anger regulation and anger reactivity as well as the self-reported situational anger level. Anger reactivity is theoretically distinct from anger regulation as emotional reactivity reflects individual differences in emotional responsiveness, whereas emotion regulation reflects the ability to modulate the emotional reaction (Mullin and Hinshaw, 2007). However, as the two constructs influence one another and have often found to be related (e.g., Kim-Spoon et al., 2013), anger reactivity served as a validation construct in the present study. Criterion validity was assessed by relating maladaptive anger regulation, assessed via behavioral observation, to measures of aggression and social rejection.

Two hypotheses were examined in our study:

Hypothesis 1 predicted that the observational measure of maladaptive anger regulation would show significant correlations with the parent- and self-reports of anger regulation and the conceptually related construct of anger reactivity. Given the features and limitations of parent- and self-reports of anger regulation outlined above, we expected the correlations between these two measures and the behavioral measure of anger regulation to be moderate in size. The correlations between the observational measure and the measures of anger reactivity and anger level were also expected to be moderate, as the latter measures reflect the construct of anger *reactivity*, which is conceptually distinct from anger *regulation*.

Hypothesis 2 postulated that the observational measure of maladaptive anger regulation would be positively associated with aggression and social rejection and make a unique contribution to the prediction of both outcomes beyond the effects of parent- and self-reports of anger regulation and anger reactivity.

## Materials and methods

### Participants

A total of 677 children aged 6–10 years were included in this study. Data from a subsample of 78 children (42 girls and 36 boys; age: *M* = 7.91, *SD* = 1.09) was used to develop and evaluate the coding system for the behavioral observation. This subsample was selected randomly from the first 250 participants. The remaining sample of 599 children (304 girls, 295 boys) provided the data for testing the validity of the observational measure. The mean age of this sample was *M* = 8.12 (*SD* = 0.92). With regard to socio-economic status, defined by the parents' educational status, 1.6% of the mothers and 1.4% of the fathers had no or a low level school qualification, 41.6% of the mothers and 48.9% of the fathers had a medium level qualification, 22.9% of the mothers and 13.6% of the fathers had university entrance qualification, and 33.9% of the mothers and 36.1% of the fathers held a university degree.

Participants were part of a larger sample of 1658 children from 33 public elementary schools who took part in a longitudinal study on intrapersonal developmental risk factors in childhood and adolescence based at the University of Potsdam in Germany. Parental consent for videotaping the children during the behavioral observation was obtained in addition to obtaining general consent to participate in the study. Only children whose parents gave permission for their child to be videotaped completed the behavioral observation task (*n* = 1183). These children did not differ significantly from those children without consent for videotaping on any of the variables included in the present study. Due to limited resources for data coding, it was not possible to analyze all videos. After excluding videos that could not be coded due to technical issues or poor light conditions (about 15%), the 677 children whose videos were included in the coding were selected randomly.

### Materials

#### Anger-eliciting task

A frustrating task designed to elicit anger was developed to assess anger regulation strategies through behavioral observation. Frustration was induced by telling the children that they could win an attractive prize if they managed to complete a task that was, in fact, almost impossible to achieve. The children were asked to build a tower out of 10 wooden toy blocks. A picture of a block tower was put in front of them, and they were instructed to build a tower that looked exactly like the tower on the picture. Three small toys and a 2:40-min hourglass were put next to the toy blocks. The experimenter sat diagonally behind the child. The children were told that they could choose one of the toys if they managed to build the tower before the hourglass had finished. The task was rigged such that two of the blocks were slightly rounded on one side. This made it almost impossible to complete the task because the tower collapsed again and again. A demonstration video showing the task is available as Supplementary Information (parental permission for including the video as Supplementary Information to this paper was obtained for the children who feature in the video). Afterwards the children were carefully debriefed by explaining to them that the task was very difficult and that hardly anyone had ever succeeded in it. All children were rewarded with a toy of their choice regardless of their performance on the task. The task was developed and pretested in a subsample of 18 children. This subsample also served to test the desirability of the presents that were offered to the children for successful performance.

#### Reports of anger regulation, anger reactivity, and anger level

As this study was embedded within a larger study, some of the questionnaires could not be used in their full length due to time constraints. The short forms used in the present study were constructed after careful theoretical considerations, as explained below. Furthermore, some of the response formats were adapted in order to keep them homogeneous across all questionnaires used in the larger study. The number of participants for whom reports were available varied from 536 to 597 between the measures (see **Table 3**).

##### Parent-reported anger reactivity

The subscale Anger/Frustration of the Temperament in Middle Childhood Questionnaire (TMCQ; Simonds, 2006) was used as a parent-report measure of anger reactivity. The TMCQ assesses temperament in children aged 7–10 years. The subscale Anger/Frustration assesses the amount of negative affect shown by the child in response to the interruption of ongoing tasks or goal-blocking (e.g., “my child gets angry when she or he has trouble with a task,” or “my child gets angry when she or he makes a mistake”). The scale consists of seven items, and the response scale ranges from 1 (*almost always untrue*) to 5 (*almost always true*). A total score was obtained by averaging the item scores. The internal consistency was α = 0.79. A bilingual speaker of English and German translated the items into German, and the accuracy was checked through back-translation.

##### Parent-reported anger regulation

Parents rated the frequency of their child's use of three anger regulation strategies: *distraction* (one item: “when my child gets angry he or she does something that he or she enjoys”), *perseveration* (one item: “when my child gets angry, what caused his or her anger won't get out of his or her mind”), and *venting the anger* (two items: “when my child gets angry he or she shows his or her anger overtly” and “when my child gets angry he or she expresses his or her anger”). These strategies were chosen because they have been found to be either negatively (*distraction*) or positively (*perseveration, venting*) related to aggression and social rejection in previous studies (e.g., Helmsen and Petermann, 2010; see Introduction). The items were derived from the Questionnaire on Emotion Regulation in Children and Adolescents (FEEL-KJ; Grob and Smolenski, 2005) and rephrased for use as parent-report items. Parents rated the frequency with which their children use these strategies when they feel angry on a 5-point scale, ranging from 0 (*never*) to 4 (*always*). A total score for the strategy *venting* was obtained by averaging across the two item scores. The internal consistency was α = 0.86. Based on the results of previous studies (see introduction), the strategy *distraction* was classified as adaptive and the strategies *perseveration* and *venting* as maladaptive. In the original classification by Grob and Smolenski (2005), the strategy *venting* was grouped into the category *other strategies* and not classified as a maladaptive strategy. However, as we defined the adaptivity of the strategies in terms of their consequences on aggression and social rejection, we treated the strategy *venting* as maladaptive. The internal consistency across all four items was α = 0.59 after recoding the scores of the items for *perseveration* and *venting the anger* The latent factor based on these items showed a good fit, as shown in **Table 4**.

##### Self-reported level of anger and sadness during the behavioral observation

Following the behavioral observation, children were asked how angry they had felt when the tower collapsed to check if the task had been successful in eliciting anger. In addition to its function as a manipulation check, the question about the anger level served as a measure for the validation of the behavioral observation as it was assumed that the anger level would be correlated positively with the use of maladaptive strategies. As the task might have elicited sadness, children were also asked about their feelings of sadness. A three-point response scale was used for both questions: 1 (*not at all*), 2 (*somewhat*), and 3 (*a lot*).

##### Self-reported anger regulation

The subscale Emotion Regulation of the Intelligence and Development Scales (IDS; Grob et al., 2009) was used to assess the children's self-report of anger regulation. Children were asked with an open-ended question what they typically do if they feel angry to get rid of their anger. If they mentioned a strategy, they were asked what else they could do. The classification of the strategies was based on the system by Grob and Smolenski (2005), with three superordinate categories: (a) adaptive strategies (e.g., *distraction, solution orientation*), (b) maladaptive strategies (e.g., *resignation, perseveration*), and (c) other strategies (e.g., *social support*). As explained above, we classified the strategy *venting the anger* as maladaptive instead of grouping it into the category *other strategies*. The children's answers were written down by the interviewer and subsequently analyzed by two trained raters, who assigned 0 points for mentioning a maladaptive strategy or no strategy at all, 1 point for mentioning a strategy of the category *other strategies*, and 2 points for mentioning an adaptive strategy, in line with Grob and Smolenski (2005). Thus, the minimum score on this measure was 0 (naming no or only maladaptive strategies), and the maximum score was 4 (naming two adaptive strategies), with higher scores reflecting more adaptive anger regulation. The answers of 134 randomly selected children were double-coded to compute the inter-rater reliability. Krippendorff's alpha was 0.80.

##### Self-reported anger reactivity

One item from the subscale Stress Management of the brief form of the BarOn Emotional Quotient Inventory: Youth Version (BarOn EQ-i:YV Brief Form; Bar-On and Parker, 2000) was used to assess children's self-report of anger reactivity (“I get angry easily”). The BarOn EQ-I assesses the emotional and social functioning of children and adolescents aged 7–18 years. The original five-point answer format was modified into a four point-scale ranging from 1 (*never*) to 4 (*often*). A bilingual speaker of English and German translated the item into German, and the accuracy was checked through back-translation.

#### Aggressive behavior

Aggressive behavior was assessed through teacher-reports of physical aggression (three items, e.g., “this child hits, shoves, or pushes peers”) and relational aggression (three items, e.g., “this child spreads rumors or gossips about some peers”). The response scale ranged from 0 (*never*) to 5 (*daily*). The items were based on the items of the Children's Social Behavior Scale—Teacher Form (CSBS-T; Crick, 1996). A total score of aggressive behavior was obtained by computing the mean score of all items. The internal consistency was α = 0.91. A bilingual speaker of English and German translated the items into German, and the accuracy was checked through back-translation.

#### Social rejection

Social rejection was assessed using teacher-, parent-, and self-report scales. The total score for each scale was obtained by summing up the item scores (after recoding items that were positively worded, so that higher scores indicate greater social rejection).

##### Teacher-reported social rejection

Teachers completed two items of the subscale Peer Relationship Problems of the teacher measure of the German version of the Strength and Difficulties Questionnaire (SDQ; Goodman, 1997; “is picked on or bullied by other children” and “is generally liked by other children”) and one self-constructed item (“is often excluded when classmates play together at break time”). The response scale ranged from 0 (*not true*) to 2 (*certainly true*). Calculating the internal consistency yielded a relatively low score of α = 0.58. However, the SDQ represents frequency counts of indicators for social rejection and is therefore not required to form an internally consistent scale.

##### Parent-reported social rejection

Three items from the subscale Peer Relationship Problems from the parent version of the SDQ were used as a parent-report measure of the children's social rejection (“is generally liked by other children,” “is picked on or bullied by other children,” and “has at least one good friend”). The response scale ranged from 0 (*not true*) to 2 (*certainly true*). The internal consistency was α = 0.67.

##### Self-reported social rejection

Five items of the subscale Social Integration of the Questionnaire on Social and Emotional Experiences at School of Elementary School Children (FEESS; Rauer and Schuck, 2003, 2004) and three items of the subscale Peer Acceptance of the German version of the Harter-Scales (Asendorpf and van Aken, 1993) were used to measure children's self-reported social rejection (e.g., “I am liked by other children,” “The other children often laugh at me”). Children indicated on a 2-point-scale whether the statements were true or not true of them (1 = *yes*, 2 = *no*). The internal consistency was α = 0.62.

### Analysis of the videotapes

The videotapes were coded using the software Eudico Linguistic Annotator (ELAN; Wittenburg et al., 2006). A coding system for the identification of regulation strategies was developed and pre-tested in an iterative process by conducting three consecutive trial codings on a subset of 20 videotapes each. Problems that occurred during the coding were successively reduced by modifying the system after each trial until a final version was reached that allowed the clear assignment of all relevant behaviors to one category. During this process, it became apparent that the strategy *distraction* had to be excluded as it turned out that the anger-eliciting situation did not offer enough opportunities for the use of this strategy. This left four maladaptive strategies (1–4), two adaptive strategies (5–6), and two further strategies (7–8) that were shown by the children but not classified as adaptive or maladaptive, as displayed in Table 1. Examples of behaviors representing the maladaptive and adaptive categories are provided in the demonstration video available as Supplementary Information.

**Table 1:** **Coding system of the behavioral observation**.

**Strategy**	**Sub-categories**	**Krippendorff's α**
1. Visual focus on the frustrating stimuli	1.1 Looking at the hourglass	0.71
	1.2 Looking at the presents	
2. Verbal focus on the frustrating stimuli	2.1 Talking negatively about the time (e.g., “time is almost up”)	0.92
	2.2 Talking negatively about the rewards (e.g., “but I want a present”)	
	2.3 Talking negatively about the tower (e.g., “it's so wobbly,” “it keeps falling”)	
	2.4 Negative self-evaluation (e.g., “I can't do it”)	
3. Venting the anger	3.1 Verbal expression of anger (swearing, e.g., “I hate this task” or “stupid tower,” grumbling)	0.73
	3.2 Anger expression (contracting the eyebrows)	
	3.3 Handling the material roughly (e.g., smashing the toy blocks on the table)	
4. Resignation	4.1 Giving up (refusing to continue for at least 3 sec)	0.99
5. Solution orientation	5.1 Testing a new strategy	0.79
	5.2 Duration of balancing	
	5.3 Working in a focused/determined way	
6. Substituting the anger expression	6.1 Smiling/laughing	0.83
7. Verbalized cognitive strategies	7.1 Positive thinking (e.g., “I can do it,” “there is still enough time”)	0.86
	7.2 External attribution: a) Attribution on insolvability of the task (“It's not my fault, it's not possible to build this tower”) b) Attribution on difficulty of the task (“It's not my fault, it's too difficult for children”)	
	7.3 Reappraisal and information seeking (e.g., “I don't care, I have enough toys at home anyway,” “Have the other kids managed to build the tower”)	
8. Ineffective help-seeking	8.1 Looking at the experimenter	0.83
	8.2 Asking for help	

The eight superordinate strategies were further differentiated into one to four sub-categories that represented observable behaviors and served as indicators for the regulation strategies. In addition to the sub-categories listed in Table 1, it was coded if the children's eyes were not clearly visible (e.g., because a child held one hand near to his or her eyes while building the tower) and if the children built the tower in a different order than prescribed. This enabled us to exclude these children from the analyses of the strategy *visual focus* (as it was not possible to determine what the child looked at; *n* = 92) or the strategy *solution orientation* (as due to the wrong order of the toy blocks the behavior *balancing*, which is a sub-category of the strategy *solution orientation*, could not be used; *n* = 24).

The videos were coded by two trained coders who were unaware of the children's aggression and peer rejection status. A subsample of 121 videos (about 20%) were double-coded to analyze the reliability of the coding system. Krippendorff's alphas, presented in Table 1, showed that three categories had an alpha below 0.80 (*visual focus on the frustrating stimuli*: α = 0.71, *venting the anger*: α= 0.73, and *solution orientation*: α = 0.79). All other categories had alphas higher than 0.80, with the highest reliability in the category *resignation* (α = 0.99). Overall, these coefficients indicate acceptable to good inter-rater reliability (Wirtz, 2006).

The sub-categories were event-coded, which means that every occurrence during the 2:40 min observation period was counted (Greve and Wentura, 1997). The scores for the strategies were calculated by summing the frequencies of the corresponding sub-categories. For two of the sub-categories of the strategy *solution orientation*, the event-sampling approach could not be used, as these categories did not reflect specific, countable behaviors. Instead, the duration of the attempt to balance the toy blocks on critical parts of the tower was measured in seconds, and the goal-orientation of the children's task performance was rated on a 4-point scale ranging from 0 (*very little engagemen*t *with the task*) to 3 (*extremely concentrated and dedicated performance*). The rating complemented the other two sub-categories, as solution-oriented behavior is a complex behavior that could not be fully captured by event-based behavioral indicators. Specific instructions regarding the coding of individual strategies are available as Supplementary Material.

### Procedure

The instruments and procedure were approved by the Ethics Committee of the authors' university as well as the Ministry of Education, Youth, and Sport of the Federal State of Brandenburg. All self-report measures and the behavioral observation task were administered in individual sessions at the school. The parent questionnaire that assessed the child's emotion regulation, emotional reactivity, and social rejection was sent home to the parents. All children received a cinema voucher and small presents for their participation. Teachers received 5 Euros for the class kitty for each completed questionnaire. After the end of the data collection period, all participating schools received a written report about the results.

### Plan of analysis

The statistical analyses were carried out with SPSS 22 and Mplus version 7.11 (Muthén and Muthén, 2012). In order to avoid reduction of the sample size, missing values were handled by the Full Information Maximum Likelihood estimation option in Mplus. To account for the non-normal distribution of the data, the robust mlr-estimator was used. All measures used in this study were analyzed as latent variables via confirmatory factor analysis except for the single-item measures (self-reported anger regulation, self-reported anger reactivity, as well as the degree of anger and sadness elicited by the task). The measurement models of the parent-reports of anger regulation and anger reactivity were specified using the corresponding items as factor indicators. The three measures of social rejection (parent-, teacher-, and self-reports) were used as indicators of a multi-informant latent factor of social rejection. The six items of aggression served as indicators of a latent factor for aggression that comprised both forms of aggression (physical and relational). To account for the shared variance of the items of the two different forms of aggression, a method factor for physical aggression was specified.

The hypotheses were tested using correlation analyses (Hypothesis 1) and structural equation modeling (Hypothesis 2). Good model fit is indicated by a comparative fit index (CFI) above 0.95, a root-mean-square error of approximation (RMSEA) below 0.06, and a standardized root-mean-square residual below 0.08 (SRMR; Hu and Bentler, 1998). A measurement model of maladaptive anger regulation, assessed through behavioral observation, was specified using the six maladaptive strategies as factor indicators: *visual focus, verbal focus, venting, resignation, (low) solution orientation*, and *(low) substitution of the anger expression*. The strategy *substituting the anger expression*, as a display rule strategy (Zeman et al., 2006), differs from the other strategies in referring to the regulation of the external expression of anger rather than the regulation of the internal experience of anger. Different authors have emphasized the importance of the conceptual and empirical distinction between these two aspects of emotion regulation (Dearing et al., 2002; Spinrad et al., 2007). However, as the use of display rules has been shown to be adaptive regarding the development of aggression and social rejection in previous studies, we still included this strategy in the measurement model in order to examine if all strategies considered to be relevant with respect to these two outcomes served as indicators for a factor reflecting maladaptive anger regulation.

As outlined in the introduction, the strategy *ineffective help-seeking* was assumed to be neither adaptive nor maladaptive in the context of the present measure. Therefore, it was not considered in the hypotheses-testing analyses. The category *verbalized cognitive strategies* contains strategies which are generally assumed to be adaptive, as they have been found to be negatively related to measures of psychopathology (e.g., Garnefski et al., 2007). However, when measured through behavioral observation, cognitive strategies can only be identified when they are verbalized. Classifying these verbalized cognitive strategies as adaptive could result in a biased assessment of the children's anger regulation skills because children who used cognitive strategies but did not verbalize them could not be identified. These children, however, might be more mature with regard to emotion regulation skills, as they have already managed to internalize their cognitive strategies (Helmsen and Petermann, 2010). Therefore, we chose not to consider these strategies in our hypotheses-testing analyses.

## Results

### Behavioral observation: descriptive statistics and bivariate correlations

The means and standard deviations of the anger regulation strategies assessed through behavioral observation are displayed in Table 2. The most frequently used strategies were *venting, visual focus*, and *substituting the anger expression*. *Resignation* had the lowest frequency. To examine gender differences, *t*-tests for independent samples were conducted rather than a MANOVA to avoid a reduction in sample size. Alpha-level adjustment for multiple testing was conducted through Bonferroni correction yielding a significance level of *p* = 0.006, and Cohen's *d* was computed as a measure of effect size. The only significant gender difference was found on the strategy *substituting the anger expression, t*_(597)_ = 3.99, *p* < 0.000, *d* = 0.33, which was more often used by girls (*M* = 5.07, *SD* = 2.87) than by boys (*M* = 4.16, *SD* = 2.94).

**Table 2:** **Means and correlations between the observed anger regulation strategies**.

	**Range**	**M (*SD*)**	**1**.	**2**.	**3**.	**4**.	**5**.	**6**.	**7**.	**8**.	**9**.
1. Visual focus	0–39	3.96 (3.60)	1	0.33^***^	0.10^*^	0.11^*^	−0.36^***^	0.01	0.18^***^	0.24^***^	−0.19^***^
2. Verbal focus	0–27	2.75 (3.54)	0.30^***^	1	0.43^***^	0.17^***^	−0.43^***^	0.22^***^	0.58^***^	0.49^***^	−0.21^***^
3. Venting the anger	0–22	4.33 (3.87)	0.10^*^	0.42^***^	1	0.14^**^	−0.27^***^	0.17^***^	0.37^***^	0.16^***^	−0.13^**^
4. Resignation	0–2	0.03 (0.19)	0.13^**^	0.18^**^	0.11^*^	1	−0.31^***^	−0.07	0.05	0.15^***^	−0.06
5. Solution orientation^a^	–	0.02 (1.60)	−0.34^***^	−0.38^***^	−0.23^***^	−0.30^***^	1	−0.14^**^	−0.35^***^	−0.40^***^	0.33^***^
6. Substituting the anger expression	0–14	4.62 (2.94)	0.04	0.21^***^	0.19^***^	−0.10^*^	−0.14^**^	1	0.20^***^	0.18^***^	−0.02
7. Verbalized cognitive strategies	0–8	1.26 (1.35)	0.20^***^	0.54^***^	0.36^***^	0.05	−0.33^***^	0.20^***^	1	0.38^***^	−0.08
8. Ineffective help-seeking	0–25	1.67 (2.35)	0.21^***^	0.50^***^	0.16^**^	0.10^*^	−0.35^***^	0.18^***^	0.35^***^	1	−0.14^**^
9. Age	6–10	8.12 (0.92)	–	–	–	–	–	–	–	–	1

*Zero-order correlations are presented above the diagonal, partial correlations controlled for age and gender are presented below the diagonal. N = 599; Exceptions: Visual focus: N = 507 (250 girls, 257 boys); solution orientation: N = 576 (293 girls, 283 boys); partial correlations: N = 486*.

a *The scores of the sub-categories of the strategy solution orientation were z-transformed prior to aggregation because of differences in response scale formats*.

* *p < 0.05*,

** *p < 0.01*,

*** *p < 0.001*.

Pearson correlation coefficients were computed to assess the bivariate associations among the strategies as well as their links with age. In addition, partial correlations, controlled for age and gender were computed. The results are displayed in Table 2 (partial correlations are presented below the diagonal). Zero-order correlations among the strategies were low to moderate, ranging from *r* = 0.01 (*visual focus* and *substituting the anger expression*) to *r* = 0.58 (*verbal focus* and *verbalized cognitive strategies*). For the majority of the categories, significant positive correlations were found. Negative correlations were found between *solution orientation* and all other strategies. The correlations with age revealed that the frequencies of *visual focus, verbal focus, venting the anger, and ineffective help-seeking* decreased whereas *solution orientation* increased with age. The partial correlations, controlled for age and gender, were very similar to the zero-order correlations.

**Figure 1:**
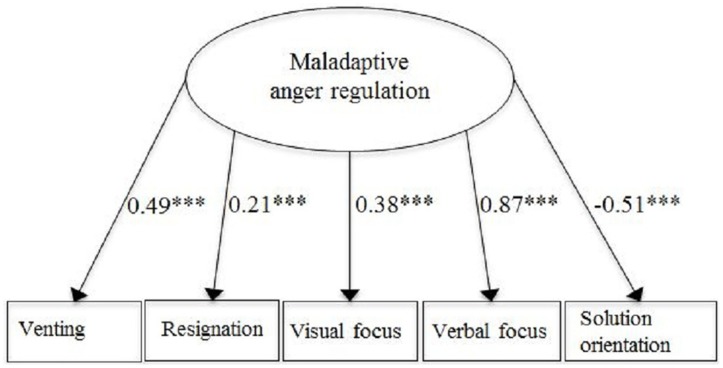
**Latent factor of maladaptive anger regulation (standardized path coefficients)**. ^***^p < 0.001; *N* = 599; Model fit: χ^2^(3) = 8.33, *p* = 0.04, RMSEA = 0.05, SRMR = 0.02, CFI = 0.99.

A measurement model with the six strategies did not fit the data well [χ^2^(9, *N* = 599) = 103.06, *p* < 0.00, RMSEA = 0.13, SRMR = 0.06, CFI = 0.79]. The factor loadings indicated that the strategy *substituting the anger expression* did not load significantly on the latent factor (β = −0.07, *p* = 0.15). This result confirmed the proposed difference between the five strategies of anger regulation and the one strategy referring to the regulation of the external expression of anger. Therefore, in a next step, we specified a measurement model excluding this strategy. This measurement model, displayed in Figure 1, showed a good fit with the data after freeing residual covariances between the indicators *solution orientation* and *visual focus* and *solution orientation* and *resignation* [χ^2^(3, *N* = 599) = 8.33, *p* = 0.04, RMSEA = 0.05, SRMR = 0.02, CFI = 0.99]. The factor-loading pattern reflected the assumed classification of the strategies: The loadings of the four strategies considered as maladaptive were positive, whereas the loading of the strategy *solution orientation*, the adaptive strategy, was negative. All factor loadings were significant at *p* < 0.001. Accordingly, this model was adopted for the further analyses.

### Validation constructs: descriptive statistics and correlations with behavioral observation

The means and standard deviations of the validation constructs, as well as their correlations with age, are displayed in Table 3. The majority of the children reported that they had experienced moderate (49.5%) or strong (40.8%) anger during the tower-building task. A minority of children (9.7%) reported they had not felt angry at all. A paired-sample *t*-test revealed that the task elicited significantly more anger than sadness, *t*_(587)_ = 16.08, *p* < 0.001, *d* = 0.66.

**Table 3:** **Means and SDs of the validation constructs and correlations with age**.

**Variable**	***N* items**	**Range**	***N***	***M* (*SD*)**	**Correlation with age**
Level of anger — self-report	1	1–3	588	2.32 (0.64)	0.03
Level of sadness — self-report	1	1–3	588	1.84 (0.72)	−0.03
Maladaptive anger regulation — parent-report					
Venting	2	1–5	561	4.14 (0.91)	−0.04
Perseveration	1	1–5	554	2.97 (1.07)	0.08
Distraction	1	1–5	552	1.91 (1.05)	0.03
Anger reactivity — parent-report	7	1–5	561	2.66 (0.73)	−0.03
Anger reactivity — self-report	1	1–4	596	2.18 (1.05)	0.08^*^
Anger regulation — self-report	1	0–4	585	1.93 (1.17)	0.11^**^
Aggression — teacher-report	6	1–5	591	1.55 (0.73)	−0.01
Social rejection — teacher-report	3	3–9	536	3.67 (1.02)	0.13^**^
Social rejection — parent-report	3	3–9	563	3.60 (0.97)	0.02
Social rejection — self-report	5	8–16	597	9.42 (1.55)	0.06

* *p < 0.05*,

** *p < 0.01*.

*T*-tests for independent samples were conducted to examine gender difference, with the significance level set at *p* = 0.004 to correct for multiple testing. There were no gender differences in the level of anger and sadness elicited by the task. The only significant difference was found on the teacher-report of aggression, *t*_(555.35)_ = −5.15, *p* < 0.00, *d* = 0.44, with boys receiving higher scores than girls (boys: *M* = 1.67, *SD* = 0.74; girls: *M* = 1.38, *SD* = 0.59). Age showed significant positive correlations with the self-report measure of anger regulation, indicating that older children reported more adaptive regulation strategies. The correlation with self-reported anger reactivity was also positive, indicating that older children more often reported to get angry easily. Furthermore, a significant positive correlation with age was found for the teacher ratings of social rejection, indicating that social rejection increased with age.

The measurement models of the validation constructs all showed a very good fit with the data (all RMSEAs < 0.05, SRMRs < 0.02, CFIs > 0.99). All fit indices as well as the factor loadings are displayed in Table 4. When modeling the parent-report factors of anger regulation and anger reactivity, the residual covariance between items that were highly similar in meaning was freed. This concerned the two items that assessed the strategy *venting* in the anger regulation questionnaire as well as items of the anger reactivity scale, which overlap in content (e.g., “Gets mad when provoked by other children and” and “Gets very angry when another child takes his/her toy away”). All indicators loaded significantly on the respective factors with *p* < 0.001. On the parent-report factor of anger regulation, the loadings of the items for *perseveration* and *venting* were positive; the loading of the *distraction* item was negative. Thus, high scores on this factor reflected maladaptive regulation. Accordingly, this factor was labeled *maladaptive anger regulation—parent-report*.

**Table 4:** **Model fits and factor loadings of the measurement models of the validation constructs**.

**Factor**	**Indicators**	**Factor loadings**	***N***	**χ^2^(*df*)**	**CFI**	**RMSEA**	**SRMR**
Maladaptive anger regulation—parent-report	Venting_1	0.62^***^	562	1.61 (1), n.s.	1.00	0.03	0.01
	Venting_2	0.55^***^					
	Perseveration	0.37^***^					
	Distraction	−0.29^***^					
Anger reactivity—parent-report	Reac_1	0.43^***^	561	22.92 (2)^*^	0.99	0.05	0.02
	Reac_2	0.61^***^					
	Reac_3	0.58^***^					
	Reac_4	0.52^***^					
	Reac_5	0.48^***^					
	Reac_6	0.65^***^					
	Reac_7	0.51^***^					
Aggression—teacher-report	Physical_1	0.58^***^	591	25.12 (6)^**^	0.99	0.07	0.01
	Physical_2	0.60^***^					
	Physical_3	0.55^***^					
	Relational_1	0.87^***^					
	Relational_2	0.88^***^					
	Relational_3	0.90^***^					
Social rejection	Teacher report	0.57^***^	599	0.98 (1), n.s.	1.00	0.00	0.02
	Parent-report	0.61^***^					
	Self-report	0.47^***^					

* p < 0.05;

** p < 0.01;

*** *p < 0.001, n.s., not significant*.

Hypothesis 1 was examined by computing partial correlations between the observational measure of maladaptive anger regulation and the validation constructs (parent- and self-reports of anger reactivity and anger regulation and self-reported anger level), controlling for age and gender. The correlations between the observational measure of maladaptive anger regulation and the validation constructs are presented in Table 5. As expected, significant, positive correlations of low to medium size were found between the observational measure and the parent- and self-reports of anger reactivity as well as the self-reported anger level during the tower-building task. However, the correlations with the parent- and self-reports of anger regulation were not significant. Thus, Hypothesis 1 was partially confirmed by the data.

**Table 5:** **Correlations between the observational measure of maladaptive anger regulation and the validation constructs**.

	**1**.	**2**.	**3**.	**4**.	**5**.	**6**.
1. Maladaptive anger regulation—behavioral observation^a^	1	0.11	0.12^*^	−0.06	0.14^**^	0.35^***^
2. Maladaptive anger regulation—parent- report^a^		1	0.73^***^	−0.15^*^	0.06	−0.05
3. Anger reactivity—parent-report^a^			1	−0.07	0.18^**^	0.10^+^
4. Anger regulation—self-report^b^				1	0.05	0.05
5. Anger reactivity—self-report^b^					1	0.13^**^
6. Situational anger level—self-report^b^						1

+ p < 0.10;

* p < 0.05;

** p< 0.01;

*** *p < 0.001*.

a Latent variable;

b *manifest variable*.

### Associations with aggression and social rejection

**Figure 2:**
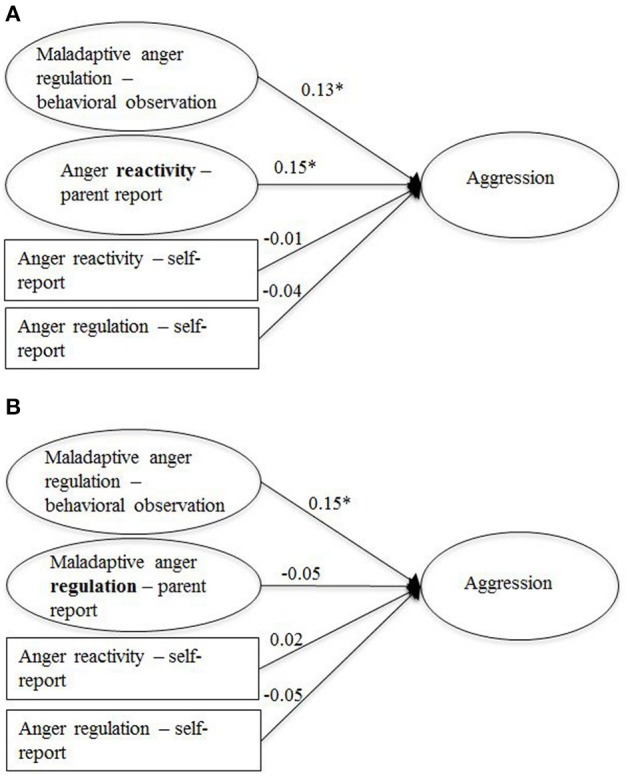
**Links between aggression and measures of anger regulation and anger reactivity (standardized path coefficients), controlled for age, gender, and anger level**. The two models differ regarding the inclusion of the parent-report measures of anger reactivity **(A)** and anger regulation **(B)**, ^*^p < 0.05, *N* = 599. **(A)** *Model fit:* χ^2^(217) = 369.08, *p* < 0.00, RMSEA = 0.04, SRMR = 0.04, CFI = 0.97; *R*^2^ = 0.04; **(B)** *Model fit*: χ^2^(157) = 275.45, *p* < 0.00, RMSEA = 0.03, SRMR = 0.03, CFI = 0.97; *R*^2^ = 0.03.

**Figure 3:**
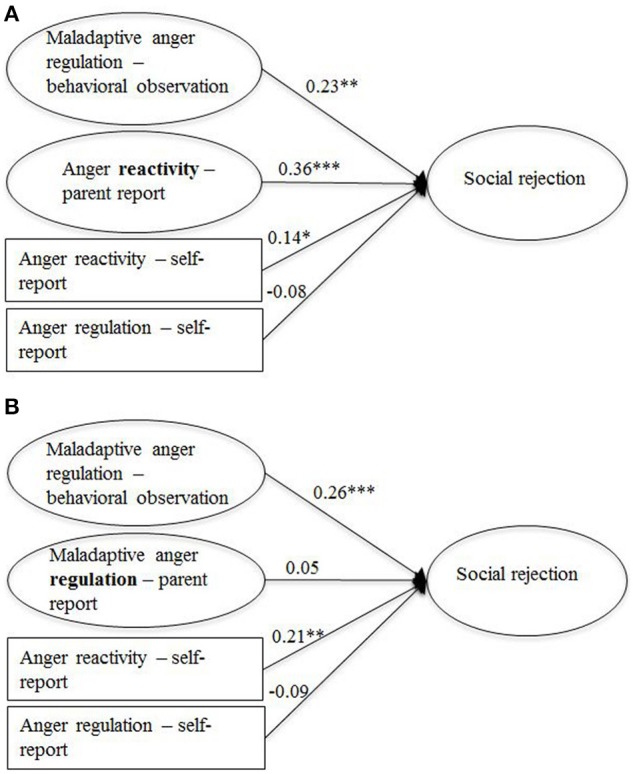
**Links between social rejection and measures of anger regulation and anger reactivity (standardized path coefficients), controlled for age, gender, and anger level**. The two models differ regarding the inclusion of the parent-report measures of anger reactivity **(A)** and anger regulation **(B)**, ^*^p < 0.05, ^**^p < 0.01, ^***^p < 0.001; *N* = 599. **(A)** *Model fit*: χ^2^(162) = 297.24, *p* < 0.00, RMSEA = 0.04, SRMR = 0.05, CFI = 0.93. *R*^2^ = 0.27. **(B)** *Model fit*: χ^2^(111) = 204.28, *p* < 0.00, RMSEA = 0.04, SRMR = 0.04, CFI = 0.93. *R*^2^ = 0.17.

Structural equation modeling was used to examine the links between the observational measure of maladaptive anger regulation and aggression as well as social rejection, proposed in Hypothesis 2. The parent- and self-reports of anger regulation and anger reactivity were included as predictors to investigate whether the observational measure made an independent contribution to the prediction of the two outcome measures. Age and gender were included as covariates. In addition, the self-reported level of anger and sadness elicited by the task were included as covariates of maladaptive anger regulation as the use of regulation strategies may have been influenced by the intensity of these two emotions. As the two parent- report measures were highly correlated (see Table 5), we did not include both variables in the same model to avoid imprecise estimations caused by multicollinearity. Instead, two separate models were computed for each outcome. The two models for aggression are presented in Figure 2A (with parent-reported anger-reactivity) and Figure 2B (with parent-reported anger regulation), the two models for social rejection are presented in Figure 3A (with parent-reported anger-reactivity) and Figure 3B (with parent-reported anger regulation). The fit for all models was acceptable or good (RMSEAs < 0.05, SRMRs < 0.05, CFIs > 0.94; see figure captions for full model fit information).

In line with Hypothesis 2, the observational measure of anger regulation made a unique contribution to the prediction of both aggression and social rejection beyond the parent- and self-report measures. The parent-reports of anger reactivity were also positively associated with both outcomes. The self-report measure of anger reactivity was linked to social rejection but not to aggression. Neither the parent- nor the self-reports of anger regulation were related to the two outcome measures.

## Discussion

The present study was designed to develop and validate an observational measure of anger regulation strategies in an anger-eliciting situation in middle childhood. Construct validity was assessed by relating the observational measure to parent- and self-report measures of anger regulation and the conceptually related construct anger reactivity. Criterion validity was examined by linking it to aggression and social rejection.

The tower-building task was successful in inducing anger in the present sample of elementary school children. Furthermore, the task elicited significantly more anger than sadness. The task takes only a few minutes to complete and does not require any special skills, which makes it suitable for administration to a large sample of children, for instance in a school setting. The coding system, developed to analyze the children's behavior during the completion of the task, allowed the comprehensive analysis of the children's anger regulation responses. Five strategies of emotional regulation were classified as maladaptive with regard to the development of aggression and social rejection (*visual focus, verbal focus, venting the anger, resignation*, and *low solution orientation*). A further strategy, *substituting the anger expression*, was initially included as a strategy referring to the regulation of the expression of anger, but was then excluded due to its failure to load on the latent factor of anger regulation.

The correlations with age revealed that older children less often focused on the frustrating stimuli (verbally and visually), vented their anger, and sought social support from the experimenter, while scoring higher on the strategy of solution orientation. Few gender differences were found, but girls more often substituted their anger expression with the expression of joy than did boys. These results are in line with previous evidence on age and gender differences in emotion regulation (Band and Weisz, 1988; Underwood et al., 1999; Zeman et al., 2006) and provide evidence for the construct validity of the observational measure.

### Construct validity

We assessed the construct validity of the latent factor of maladaptive anger regulation based on the behavioral observation by examining its correlations with three pertinent constructs: (a) anger regulation (parent- and self-reports), (b) anger reactivity (parent- and self-reports), and (c) self-reported anger level during the task (assuming that the more anger the task elicited, the more likely it would be that children engaged in maladaptive regulation strategies). The use of maladaptive strategies in response to the anger-eliciting task was significantly correlated with higher parent-rated and self-reported anger reactivity, and with greater self-reported anger during the behavioral observation. As expected, the correlations were moderate in size, which supports the conceptualization of emotional reactivity and emotion regulation as interrelated, but conceptually distinct constructs (Rothbart and Sheese, 2007). No significant correlations were found with parent- and self-reported anger regulation.

One possible explanation for the non-significant correlation of parents' assessment of anger regulation with the observational measure is that parents' ratings are largely limited to their children's behavior within the family context. The behavioral observation task may have evoked less anger display due to the presence of an unfamiliar experimenter and the awareness of being videotaped. The behavior during the tower-building task may more closely reflect the children's behavior within the school setting than their behavior in the family context as in the school-setting children are likely to be more concerned about the consequences of venting their anger openly. Another explanation may lie in the high correlation between the parent-ratings of anger reactivity and anger regulation found in the present study. Theoretically, a child with high anger reactivity can be skilled in anger regulation and vice versa. The high correlation indicates that the parents found it difficult to differentiate between the two constructs, which suggests that parents may not be a good source of information on anger regulation unconfounded by anger reactivity.

In conclusion, the proposed links of observed anger regulation with parent- and self-reports predicted in Hypothesis 1 were partly confirmed by the data. The lack of significant associations of observed maladaptive anger regulation strategies with parent-rated maladaptive regulation and self-reported anger regulation skills may to some extent reflect the limitations of parent- and self-reports of anger regulation, outlined in the introduction. Children in the present age group may be too young to give valid self-reports of anger regulation, and—as suggested by previous research—their self-reports of anger regulation may not correspond to their actual behavior in a real situation. Parents may be unable to differentiate between anger reactivity and anger regulation. In combination, these problems call for alternative methods for assessing anger regulation, such as behavioral observation. However, our results do not undermine the importance of parent and self- reports *per se*. Parent-reports can provide important data about the children's anger regulation at home, particularly about the external anger-related behavior. Self-reports provide valuable insights about the children's theoretical knowledge about regulations strategies. In addition, the self-report measure offers the opportunity to report internal cognitive strategies, which, as they are not observable, cannot be assessed through either behavioral observation or parent ratings. The differential suitability of the methods for assessing different anger regulation strategies highlights the importance of a multi-method approach to capture a broad range of the children's use of regulation strategies.

### Criterion validity

In line with Hypothesis 2, we found that the observational measure of maladaptive anger regulation was significantly linked to aggression measured by teacher-reports, and social rejection assessed by self-, parent-, and teacher-reports. These findings support the criterion validity of the observational measure as they are consistent with a large number of studies that also have found that children with deficits in anger regulation are rated as more aggressive and are more socially rejected than children with more adaptive regulation skills (see Lemerise and Harper, 2010, for a review). With regard to aggression, this link can be explained by the action tendency associated with anger, as this action tendency is assumed to activate aggression-related motor impulses (Berkowitz and Harmon-Jones, 2004). Accordingly, the likelihood of aggression is increased for children who use maladaptive anger regulation strategies, as these strategies do not effectively reduce the intensity and frequency of angry feelings. With regard to social rejection, our results support the notion that maladaptive forms of anger regulation may irritate peers and disturb ongoing peer interactions, leading to social rejection. In addition, low use of solution-oriented behavior may be associated with the inability to constructively solve conflicts with peers (Maszk et al., 1999).

Our results suggest that the observational measure may be more valid compared to the parent- and self-report measures of anger regulation in the present age group, as neither the parent-report nor the self-report measure were linked to aggression or social rejection.

Further evidence for the validity of the observational measure was provided by the fact that maladaptive regulation, assessed through observation, was uniquely linked to both aggression and social rejection. The significant association of observed maladaptive anger regulation with social rejection held when controlling for both self-reported and parent-reported anger reactivity, and the association with aggression held over and above a significant link with parent-reported anger reactivity. This result is in line with previous studies that have found that anger reactivity and anger regulation predict unique variance in outcome measures such as externalizing behavior problems and social functioning (Eisenberg et al., 1995, 2005).

### Strengths and limitations

We believe our study has several strengths. We employed a realistic anger-eliciting task and developed a reliable coding system for identifying maladaptive strategies of anger regulation. The task is suitable for administration in short school-based testing sessions and can therefore be used economically in large samples of children. The observational measure was compared to information obtained from the children and their parents on habitual anger regulation and anger reactivity to establish its construct validity. Moreover, we demonstrated the criterion validity of the observational method through relating it to measures of aggression and social rejection, also using data from multiple informants.

At the same time, some limitations of our study have to be mentioned. The stability of the children's anger regulation strategies in a similar task needs to be tested in future research. The generalizability of the behavior shown during the behavioral observation also remains to be tested, as the children were observed in an arranged situation that, to some extent, constrained their opportunities to act. For example, children had very limited opportunities to distract themselves from the anger-eliciting task. Therefore, as noted above, the strategy *distraction* could not be assessed through the observational measure, although it is likely that some children might have used this strategy in a natural situation. This limitation may also serve to explain why the behavioral observation measure did not correlate with the parent- and self- reports of anger regulation, as parents and children may have thought of different situations than the one assessed with the observational measure. Similarly, the presence of an unresponsive experimenter who did not provide support meant that *seeking social support*, considered adaptive in other situations, was classified as neither adaptive nor maladaptive in the present measure.

In addition, as we assessed only one adaptive strategy, namely *solution orientation*, we were not able to examine the link between the number of strategies a child uses and aggression and social rejection. Using one regulation strategy at a high level may be less adaptive than using moderate levels of several strategies, as suggested by previous findings that children who use various adaptive strategies are less aggressive than children who use just one (Gilliom et al., 2002; see also Lougheed and Hollenstein, 2012, for a similar finding with regard to internalizing problems).

Finally, the results regarding the parent-ratings of anger regulation may have been affected by the fact that we were unable to include the selected scales in full and had to adapt the items slightly for use as a parent-report measure.

Despite these limitations, our study contributed to the existing literature on the assessment of anger regulation in children by providing an easily applicable observational method for the assessment of anger regulation strategies in middle childhood. It further showed that maladaptive regulation, assessed with this new measure, contributed independently to the prediction of aggression and social rejection beyond the effect of parent- and self-reports of anger regulation and anger reactivity. Thus, our observational measure is recommended as part of a multi-method approach to studying anger regulation in childhood in which the strengths of different methods complement each other. For example, our results indicate that compared to self-reports, observational measures are better able to assess the behavior in a real anger-eliciting situation. Self-reports, on the other hand, may be more suitable for assessing the children's theoretical knowledge about emotion regulation. The results of our study provided insights about the advantages and limitations of parent-reports, self-reports, and observational measures that may be helpful for future research on anger regulation in middle childhood.

## Author contributions

Both authors have contributed substantially to the conception and design of the work as well as to the analysis of the data. HR has primarily collected, analyzed, and interpreted the data, BK has provided input and supervision to the analyses and writing up of the study. Both authors agreed to be accountable for all aspects of the work.

### Conflict of interest statement

The authors declare that the research was conducted in the absence of any commercial or financial relationships that could be construed as a potential conflict of interest.
