# Supplementary material for: Assessing anger regulation in middle childhood: development and validation of a behavioral observation measure
Source: Front Psychol. 2015 Apr 24;6:453. doi: 10.3389/fpsyg.2015.00453 (PMC4408751; doi:10.3389/fpsyg.2015.00453)
Supplement: Supplementary file 1 [file Table1.PDF]

# *Supplementary Material*

# Assessing anger regulation in middle childhood: development and validation of a behavioral observation measure

**Helena Rohlf*, Barbara Krahé**

University of Potsdam, Department of Psychology, Potsdam, Germany

*** Correspondence:** Helena Rohlf, University of Potsdam, Department of Psychology, Karl-Liebknecht-Str. 24-25, 14476 Potsdam, Germany.
helena.rohlf@uni-potsdam.de

## 1. Supplementary Data

A demonstration video showing the anger-eliciting task is available as supplementary information (parental permission for including the video as supplementary information to this paper was obtained for the children who feature in the video).

## 2. Supplementary Table

**Supplementary Material: Table 1 | Instructions for the coding of the video data.**

| **Strategy** | Specific Instructions |
|---|---|
| Visual focus on the frustrating stimuli | The sub-category *looking at the hourglass* is not coded at the very beginning when the experimenter turns the hourglass around. |
| Verbal focus | Questions that are obviously not directed at the experimenter but have rhetorical character, and that refer to the negative aspects of the task, are coded in this category (e.g. “How is this supposed to work”, “Why do I have to do such a difficult task?”) |
| Venting the anger | In the sub-category *verbal expression of anger*, only *angry* expressions are coded; expressions that indicate sadness or disappointment are not coded. |
| Resignation | Is coded if the child stops building the tower for at least three seconds without showing any engagement with the task; typical indicators: child crosses arms, leans back; is not coded when the child stops working on the task to talk to the experimenter. |
| Solution orientation | The sub-category *balancing* refers to the attempt to equilibrate the rounded toy blocks or the part above the rounded toy blocks with the aim to make the tower stand unsupported:<br>- is only coded if the duration is at least one second<br>- is not coded of the child is not looking at the construction<br>- is not coded if the child is only holding but not actively equilibrating the blocks<br>The sub-category *using an alternative approach* refers to the attempt to build the upper section of the tower first and then placing it on top of the lower section in one part. |
| Substituting the anger expression | *Laughing/smiling* is coded dependent on the context (→ is not coded if a child is happy because he or she has almost finished the tower). |

| | |
|---|---|
| Verbalized cognitive strategies | Comments referring to the difficulty or insolvability of the task are coded in the strategy *verbalized cognitive strategies* only for the first time they were mentioned. All following comments with the same meaning are coded in the category *verbal focus on the frustrating stimuli* as insisting that the task is difficult or insolvable reflects the strategy of focusing on the negative characteristics of the situation. |
| Ineffective help-seeking | *Looking at the experimenter* is not coded if the child looks at the experimenter because the experimenter picks up a dropped toy block |
| **General coding instructions** | |
| Specific situations | Behaviors that are caused by events that are unrelated to the task (e.g. bell rings, person enters the room) are not coded. |
| Verbal comments | Comments that are unrelated to the task and comments that are related to the task but cannot be classified into one of the categories are coded into an extra category, named *other comments* (e.g. “Is the camera switched on?”). |
| Practical implementation of the coding | The sub-categories are classified into five groups. Each group includes behaviors that can easily be coded concurrently. Thus, each video is coded in five runs. In each run the rater has to pay attention to only one of the following groups of behaviors:<br>1) Gaze<br>2) All kinds of verbal utterances<br>3) Facial expression<br>4) All behaviors that directly involve the toy blocks (e.g. balancing, alternative approach) + *resignation*<br>5) Extent to which a child works in a focused/determined way (rating) |
